# Occupational Therapy–Based Physical Activity Questionnaire (OTBPAQ) Categorization of Physical Activity Composition Ratios of Older Individuals Living in the Community

**DOI:** 10.1155/oti/3980869

**Published:** 2025-12-31

**Authors:** Daisuke Tashiro, Masahiro Ogawa, Makoto Otaki, Toshimichi Nakamae, Takako Morikawa, Jumpei Oba

**Affiliations:** ^1^ Department of Occupational Therapy, Faculty of Rehabilitation, Kobe Gakuin University, Kobe, Hyogo, Japan, kobegakuin.ac.jp

**Keywords:** frailty, Kihon checklist, occupation, physical activity

## Abstract

**Background:**

This study is aimed at calculating and categorizing the composition ratios of physical activity categories in relation to total daily physical activity among older adults living in a community using the Occupational Therapy–Based Physical Activity Questionnaire (OTBPAQ) and at examining their characteristics.

**Participants and Methods:**

The survey used the OTBPAQ to evaluate the composition of physical activity categories as well as a questionnaire on participant characteristics (sex, age, presence of family members, subjective well‐being, hobbies and activities, physical pain, frailty, and physical activity). The OTBPAQ was developed as an evaluation chart concerning the “occupation” category. Subsequently, cluster analysis was conducted based on the composition ratios of physical activity categories in relation to total daily physical activity, and the relevance of the participant characteristics was confirmed for each extracted group.

**Results:**

A total of 499 participants were allocated using cluster analysis into five groups, namely, “balance‐oriented group,” “aerobic exercise group,” “shopping group,” “sports group,” and “recreational gymnastics group,” based on the main activity categories of each cluster. Results showed that participant characteristics, such as the amount of physical activity and number of physical activity categories, differed among the clusters.

**Conclusion:**

The OTBPAQ could extract five clusters of older adults in a community based on the composition ratio of physical activity categories. Examining the composition ratio of physical activity categories can facilitate a highly individualized approach to physical activity for each group, leading to more effective care prevention measures to increase healthy life expectancy.

## 1. Introduction

With the increased global population of older adults in recent years, extending their healthy life expectancy has become a significant goal. Physical activity is closely related to healthy life expectancy and has been shown to prevent cardiovascular symptoms [1, 2], reduce the risk of fall‐related fractures [3], maintain or improve physical fitness [4, 5], and prevent frailty [6].

Physical activity consumes more energy than the resting state. It is broadly classified into two categories: “instrumental activities of daily living (IADL)” such as work, housework, and commuting to and from work and “exercise” that is performed in a planned and continuous manner to maintain and improve physical fitness, including physical fitness related to sports competitions and health [7].

The amount of physical activity is generally evaluated by “subjective evaluation,” in which the participants are asked to look back on their daily lives and indicate the time spent performing physical activity corresponding to each intensity (light, medium, and high intensity), or by “objective evaluation” using a physical activity meter or pedometer. Since these methods can quantitatively evaluate the amount of physical activity, they often express the amount of physical activity using the unit of metabolic equivalent for tasks (METs)‐hours/week by simultaneously obtaining information on the duration and intensity of physical activity. One MET is calculated by dividing the work metabolic rate by the resting metabolic rate. In Japan, the Ministry of Health, Labour and Welfare [7] has established standards for the amount of physical activity required for each life stage, and increasing the amount of physical activity is crucial for extending healthy life expectancy.

Physical activity can be seen as a comprehensive activity that has a multifaceted impact on physical functions [8, 9], cognitive functions [10], sociality [11], and exercise. For this reason, it is not enough to discuss the total amount of physical activity among older adults, but the proportion of each physical activity category (i.e., the composition ratio of physical activity categories in relation to total daily physical activity) must also be examined. The amount of physical activity must be increased by customizing the physical activity categories within the time constraints of environmental and individual factors, as well as the individual’s physical strength. It is essential to evaluate the “composition ratio of the physical activity categories” of each individual and provide interventions tailored to the individual’s lifestyle to lead a community life. However, adequate surveys from this perspective are yet to be conducted.

According to the 2021 Basic Survey on Social Life conducted by the Japanese Ministry of Internal Affairs and Communications, the percentage of people aged 75 and over who engage in physical activity is 53.7% for sports; 7.2% for hobbies and recreation; 23.3% for learning, self‐development, and training; and 16.4% for volunteer activities [12], showing that many older adults participate in some activity. However, this is only the percentage of older people who are physically active, and it is also assumed that among those who are physically active, there are naturally also people who are judged to be “frail” on questionnaires that ask about the impact of various factors, such as the Kihon checklist (described below) [13]. For this reason, in addition to the amount and content of physical activity, it is necessary to classify these activities according to the intensity and frequency of the composition ratio of physical activity.

Older adults living in the community are categorized into activity groups based on the amount of physical activity and the composition ratio of the physical activity categories relative to total physical activity. By dividing older adults into groups based on this categorization, we can consider highly individualized approaches to physical activity that match the characteristics of each group, and this meaningful survey could lead to new intervention strategies.

Therefore, we developed the Occupational Therapy–Based Physical Activity Questionnaire (OTBPAQ), which assesses the composition of physical activity across a wide range of physical activity categories. Based on occupational therapy, the OTBPAQ is expected to extract physical activity categories and the amount of physical activity from a wide range of daily activities. This study is aimed at calculating and classifying the composition ratios of physical activity categories for older people living in the community using OTBPAQ and examining their characteristics. This would allow us to explore whether the composition ratios of physical activity are related to healthy life expectancy indicators, such as the prevention of frailty.

## 2. Material and Participants

### 2.1. Participants

The study participants were older residents of Akashi City who attended a community center for older individuals. The survey was conducted from September 5 to December 22, 2022, in cooperation with the Akashi City Welfare Bureau Community and Symbiotic Society Office. The researchers visited each community center to explain the study and provided instructions on how to respond. Explanations were added as required according to the level of understanding of participants.

### 2.2. Survey Contents

The survey consisted of a self‐administered questionnaire on the OTBPAQ, Kihon checklist, and participant characteristics.

#### 2.2.1. OTBPAQ

**Figure 1:**
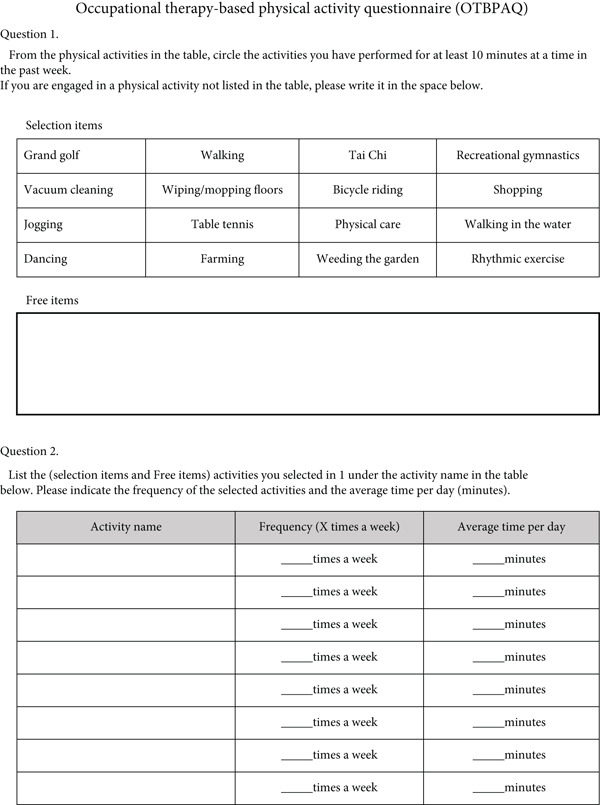
Occupational therapy–based physical activity questionnaire (OTBPAQ).

The OTBPAQ was developed by Tashiro et al. to identify the composition of occupational therapy–based physical activity categories relative to total physical activity. Here, “occupational therapy–based physical activity categories occupational therapy–based physical activity categories” refers to the nine activities essential for daily living (ADLs, IADLs, health management, rest and sleep, education, work, play, leisure, and social participation). OTBPAQ focuses on IADLs with high physical demands. It is a self‐administered questionnaire with 16 items covering physically demanding IADLs performed in the past week; unlisted items can be added in the free response section. Participants report the frequency and duration of these activities (Figure 1). The items are based on a list of evaluation indices used in the occupational therapy field, such as the Functional Independence Measure, Frenchay Activities Index, Tokyo Metropolitan Institute of Gerontology, Index of Competence, Canadian Occupational Performance Measure, and Aid for Decision‐making in Occupation Choice. The items were extracted based on the following conditions: (1) moderate‐to‐vigorous intensity (≥ 3.0 METs) and (2) high‐frequency activity, which refers to activities that the majority of older adults frequently perform in their daily lives. The items were classified by four occupational therapists who had been working in the field for more than 10 years, considering the categories of “occupation” in the occupational therapy field of domain and process [15]. In addition to the classification by the composition ratio of physical activity categories obtained from the OTBPAQ, the amount of physical activity (low: < 10 MET‐hours/week, moderate: 10.0–49.9 MET‐hours/week, and high: ≥ 50 MET‐hours/week [16]) and the number of physical activity items were calculated.

#### 2.2.2. Kihon Checklist

**Figure 2:**
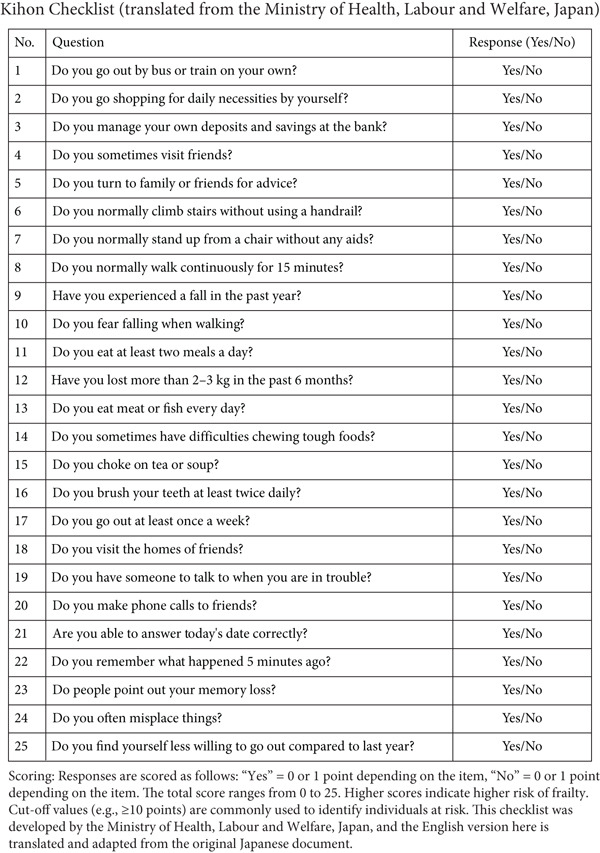
Kihon checklist (translated from the Ministry of Health, Labour and Welfare, Japan).

The Kihon checklist is a self‐administered questionnaire consisting of 25 questions in seven domains, namely, ADL, locomotion, low nutritional status, oral function, confinement, cognitive function, and depressive mood [13] (Figure 2). For each of these domains, the selection criteria for prevention support participants were determined, and the validity of the selection criteria was verified in an epidemiological study of older adults living in the community [17]. Among the selection criteria, the risk of loss of independent function was the highest when 10 or more of the 20 items, excluding depressed mood, were present, suggesting the importance of multidimensional evaluation. In this study, only 20 items, excluding depressed mood, were investigated. Those with five or more of these 20 items were classified as frail [18].

#### 2.2.3. Participant Characteristics

Data on sex, age, family members living with the patient, subjective well‐being (5‐point scale: 1, *good*; 2, *fair*; 3, *normal*; 4, *not so good*; and 5, *bad*), hobbies, and physical pain were collected. The amount of physical activity and the number of physical activity items obtained from the OTBPAQ were included as the participant characteristics.

## 3. Method

The OTBPAQ was analyzed based on the following methods.

### 3.1. Calculation of Physical Activity

The MET values for each physical activity category extracted from the OTBPAQ were selected based on those reported by Willis et al. in “Energy costs of human activities in adults aged 60 and older (Older Adult Compendium of Physical Activities)” [19]. When no corresponding activity was listed in the Older Adult Compendium of Physical Activities, the MET value was determined based on the activity judged most similar by six occupational therapists (D.T., M.O., M.O., T.N., T.M., and J.O.). The METs for each physical activity category were multiplied by the weekly frequency (days/week) and average daily duration (hours/day) to calculate the amount of physical activity per week (MET‐hours/week) for each category.

### 3.2. Classification of Physical Activity Categories Based on the Occupation

The selected physical activity categories were classified into one of nine categories based on occupational therapy categories: IADL, health management, rest and sleep, education, work, play, leisure, and social participation [14, 15].

Additionally, while classifying them into nine occupational therapy categories, they were further subdivided into subcategories to enable detailed identification. Note that the above classification was performed by six occupational therapists (D.T., M.O., M.O., T.N., T.M., and J.O.) based on the similarity of the physical activity content.

### 3.3. Statistical Analysis

We calculated the composition ratios of physical activity MET‐min/week to the total physical activity MET‐min/week ([subcategory MET − min/week divided by total physical activity MET − min/week] × 100) and then conducted cluster analysis. Clusters were stratified using the Ward method, and the Euclidean square distance was used to determine the distance between groups. The number of clusters was determined from the dendrogram. Prior to the group comparisons, the normality of all continuous variables was assessed using the Shapiro–Wilk test, and the subsequent statistical analyses were selected according to the normality results. Consequently, to clarify the characteristics of the clusters, the relationships between the extracted clusters and participant characteristics (sex, age, presence of family members, subjective well‐being, hobbies, physical pain, frailty, amount of physical activity, and number of physical activity items) were examined by *χ* ^2^ test, one‐way analysis of variance (ANOVA), and Kruskal–Wallis test. Comparisons between clusters were made using residual analysis with adjusted standardized residuals for the *χ* ^2^ test (an absolute value of 1.96 or greater was considered significant). Statistical Package for Social Sciences (Windows Version 26.0, International Business Machines Corp., Armonk, New York, United States) was used for statistical analyses. The significance level was set at 5%.

### 3.4. Ethical Considerations

The purpose and significance of the study and the management of personal information were explained to the study participants, and written consent was obtained before the questionnaire survey was administered. This study was approved by the Faculty of Comprehensive Rehabilitation Studies Ethics Committee at Kobe Gakuin University (Approval No. SORIN21‐08) and was conducted in accordance with the Declaration of Helsinki.

## 4. Results

### 4.1. Participant Information

Of the survey participants, 499 (69 males [13.8%] and 430 females [86.2%]) were included in the analysis, and 26 participants who did not engage in physical activity of 3.0 METs or more during the study period were excluded from the analysis. The mean age of the participants in the analysis was 79.2 years (standard deviation [SD] = 6.05, range 65–95 years). There were 127 participants (25.5%) aged 65–74 years and 372 participants (74.5%) aged 75 years or older. The average age of male participants was 81.5 years (SD = 5.80), and the average age of female participants was 78.0 years (SD = 6.02). There was no significant difference in age between the sexes (Table 1). Table 2 shows the distribution of physical activity levels over the week. For physically active participants, 51 (10.2%) engaged in physical activity of 3.0–10 MET‐hours/week, and 448 (90.8%) engaged in physical activity of ≥ 10 MET‐hours/week.

**Table 1:** The age distribution of study participants (*n* = 499).

	**Male**	**Female**	**Total**
*n* (%)	69 (13.8)	430 (86.2)	499 (100)
65–74	13 (18.8)	114 (26.5)	127 (25.5)
75–	56 (81.2)	316 (73.5)	372 (74.5)
Age (SD)	81.5 (5.80)	78.0 (6.02)	79.3 (6.1)

*Note:* Twenty‐six participants who did not engage in physical activity of ≥ 3.0 METs during the study period were excluded from the analysis.

**Table 2:** Distribution of total MET‐hours/week of physical activity among study participants.

**MET-hours/week**	**n** **(%)**	**Sex (male/female)**	**Age (SD)**
Total	499 (100)	69/430	79.3 (6.1)
3–9	51 (10.2)	7/44	81.1 (6.1)
10–22	119 (23.8)	7/112	78.8 (6.1)
23–49	206 (41.3)	15/191	79.3 (6.1)
50–99	95 (19.0)	27/68	78.1 (6.1)
100–	28 (5.6)	3/25	79.3 (6.2)

*Note:* MET‐hours/week is computed as the ∑(METs × hours per week) for each activity reported on the OTBPAQ.

### 4.2. Classification of Physical Activity Based on the Occupation

Table 3 shows the physical activity categories and the number of participants engaged in each category based on the results of the OTBPAQ survey. Of the 39 physical activity categories, IADLs, health management, work, and social participation were the most similar among the occupational therapy categories. For the subcategories, IADLs were selected as “cleaning,” “gardening,” and “shopping”; health management as “strength exercise,” “aerobic exercise,” and “recreational gymnastics”; and work as “social participation.” Work was categorized as one item of “productive activity” and social participation as one of “sports.”

**Table 3:** Occupational therapy–based classification of physical activities recalled on the OTBPAQ (*n* = 499).

**Occupation category (9 items)**	**Subcategory (8 items)**	**METs**	**Physical activity items (39 items)**
Activities of daily living (ADLs)			

Instrumental activities of daily living (IADL)	Cleaning (*n* = 471)	4.3	Vacuum cleaning (*n* = 273)
		3.5	Wiping/mopping floors (*n* = 195)
		4.3	Bath cleaning (*n* = 2)
		4.3	Use the mopping (*n* = 1)
	Gardening (*n* = 168)	4.8	Weeding the garden (*n* = 164)
		4.8	Flower care (*n* = 1)
		4.8	Vegetable garden (*n* = 3)
	Shopping (*n* = 404)	3.8	Shopping (*n* = 403)
		5.3	Bicycle riding and shopping (*n* = 1)

Health management	Strength exercise (*n* = 51)	5.0	Exercise at gym (*n* = 2)
		5.0	Strength training (*n* = 4)
		5.0	Stretching and strength training (*n* = 1)
		5.0	Training gym (*n* = 1)
		3.5	Training goods (*n* = 1)
		3.8	Tai Chi (*n* = 20)
		3.8	Yoga (*n* = 7)
		4	Walking in the water (*n* = 11)
		5.3	Swimming (*n* = 4)
	Aerobic exercise (*n* = 446)	4.0	Walking (*n* = 282)
		4.0	Stepper (*n* = 1)
		4.5	Exercise while walking (*n* = 1)
		7.3	Jogging (*n* = 15)
		5.3	Bicycle riding (*n* = 146)
		6.3	Exercise bike (*n* = 1)
	Recreational gymnastics (*n* = 424)	3.5	Recreational gymnastics (*n* = 317)
		3.8	Rhythmic exercise (*n* = 106)
		3.5	Hula hoop (*n* = 1)

Rest and sleep			

Education			

Work	Productive activity (*n* = 40)	4.8	Farming (*n* = 30)
		5.0	Physical care (*n* = 8)
		3.3	Volunteer activities (*n* = 1)
		4.8	Community cleanup (*n* = 1)

Play			

Leisure			

Social participation	Sports (*n* = 92)	3.5	Table tennis (*n* = 22)
		4.3	Grand golf (*n* = 56)
		4.3	Golf (*n* = 2)
		6.0	Dancing (*n* = 5)
		6.3	Folk dance (*n* = 1)
		6.3	Traditional dance club (*n* = 4)
		5.3	Tennis (*n* = 1)
		3.5	Fishing club (*n* = 1)

*Note:* The occupation category was extracted from the “occupation” category in the domain and process occupational therapy domain developed by the American Occupational Therapy Association (2014). Physical activity items were extracted from the OTBPAQ results. METs for each physical activity category extracted from the OTBPAQ were selected based on the METs in the Older Adult Compendium of Physical Activities by Willis et al. [14]. Subcategories were created and categorized so that specific categories could be identified for each item.

### 4.3. Cluster Classification

**Figure 3:**
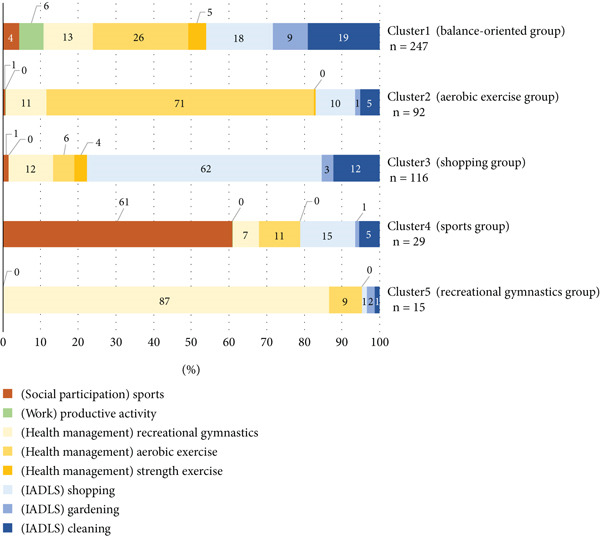
Characteristics of each of the five cluster classification groups (*n* = 499). The graphs indicate the percentage (%) of each category of total physical activity. IADL, instrumental activities of daily living.

Cluster classification was conducted based on the percentage of physical activity (MET‐hour/week) in the eight “occupational therapy categories” subcategories described above. Five clusters were extracted from the cluster analysis. The results are shown in Figure 3. Cluster 1, “balance‐oriented group,” included 247 (49.5%); Cluster 2, “aerobic exercise group,” included 92 (18.4%); Cluster 3, “shopping group,” included 116 (23.2%); Cluster 4, “sports group,” included 29 (5.8%); and Cluster 5, “recreational gymnastics group,” included 15 (3.0%). These clusters were named after the subcategories that tended to have a high proportion of the eight subcategories.

### 4.4. Participant Characteristics of Each Cluster

To clarify the participant characteristics of each cluster, analyses were first conducted for the essential attributes of sex, age, and family members living together. The results showed that there was a significant difference based on sex (*p* < 0.05), and residual analysis revealed a significantly higher percentage of males in the “aerobic exercise group” and “sports group.” However, there were no significant differences based on age or type of residence (Table 4).

**Table 4:** Cluster membership by participant characteristics (*n* = 499).

**Variable**	**Answer category**	**Total (** **n** = 499**)**	**Cluster 1 (** **n** = 247**)**	**Cluster 2 (** **n** = 92**)**	**Cluster 3 (** **n** = 116**)**	**Cluster 4 (** **n** = 29**)**	**Cluster 5 (** **n** = 15**)**	**Analysis**	**p** **value** **Adjusted SD** ^∗^ **1** **Bonferroni** ^∗^ **3**
		**—**	**Balance-oriented group**	**Aerobic exercise group**	**Shopping group**	**Sports group**	**Recreational gymnastics group**		
Sex	Male	69 (13.8)	28 (11.3)	19 (20.7)	12 (10.3)	9 (31.0)	1 (6.7)	^∗^1	0.008 ^∗∗^
			−1.60	2.10	−1.24	2.77	−0.82		
	Female	430 (86.2)	219 (88.7)	73 (79.3)	104 (89.7)	20 (69.0)	14 (93.3)		Cl1,3,5 < Cl2, 4†
			1.60	−2.10	1.24	−2.77	0.82		

Age (years old)		79.3 (6.1)	79.2 (5.9)	79.2 (6.3)	78.6 (6.2)	80.0 (5.7)	81.2 (7.3)	^∗^2	n.s.	

Presence of family members	Yes	306 (61.3)	159 (64.4)	55 (59.8)	66 (56.9)	18 (62.1)	8 (53.3)	^∗^1	n.s.	
	No	193 (38.7)	88 (35.6)	37 (40.2)	50 (43.1)	11 (37.9)	7 (46.7)		

Subjective well‐being			3 (2–3)	3 (2–3)	3 (3–4)	3 (2–3)	3 (3–4)	^∗^3	n.s.	
			246.0	247.9	258.9	232.9	293.0		

Pleasure activities	Yes	436 (87.4)	210 (85.0)	80 (87.0)	103 (88.8)	29 (100.0)	14 (93.3)	^∗^1	n.s.	
	No	63 (12.6)	37 (15.0)	12 (13.0)	13 (11.2)	0 (0.0)	1 (6.7)		

Physical pain	Yes	306 (61.3)	157 (63.6)	51 (55.4)	71 (61.2)	17 (58.6)	10 (66.7)	^∗^1	n.s.	
	No	193 (38.7)	90 (36.4)	41 (44.6)	45 (38.8)	12 (41.4)	5 (33.3)		

Frailty	Frailty	206 (41.3)	96 (38.9)	28 (30.4)	56 (48.3)	15 (51.7)	11 (73.3)	^∗^1	0.005 ^∗∗^	
			−1.09	−2.34	1.75	1.18	2.56		
	Robust	293 (58.7)	151 (61.1)	64 (69.6)	60 (51.7)	14 (48.3)	4 (26.7)		Cl2 < Cl1,3,4 < Cl, 5†	
				1.09	2.34	−1.75	−1.18		−2.56	

Amount of physical activity (MET‐hours/week)	High ≥ 50	123 (24.6)	74 (30.0)	27 (29.3)	13 (11.2)	9 (31.0)	0 (0.0)	^∗^1	< 0.001 ^∗∗∗^	
			2.72	1.16	−3.83	0.82	−2.25		
	Moderate/low 10.0–49.9/< 10	320/56 (64.1/11.2)	152/21 (61.5/8.5)	57/8 (62.0/8.7)	85/18 (73.3/15.5)	19/1 (65.5/3.5)	7/8 (46.7/53.3)		Cl3, 5 < Cl2, 4 < Cl1†	
				−2.72	−1.16	3.83	−0.82		2.25	

Number of physical activity items			5 (4–6)	4 (3–5)	3 (2–4)	4 (3–6)	2 (1–3)	^∗^3	< 0.001 ^∗∗∗^	
			247.0	116.0	92.0	29.0	15.0		Cl4 < Cl1^∗∗∗^,2 ^∗∗^ Cl5 < Cl1,2,3^∗∗∗^	

*Note:* The tests used were the *χ* ^2^ test ( ^∗^1), one‐way analysis of variance ( ^∗^2), and Kruskal–Wallis test ( ^∗^3). In the analysis of  ^∗^1, “number of persons (%)” is shown in the upper row, “adjusted standard deviation” in the lower row, “mean (standard deviation)” in  ^∗^2, and “median (first quartile–third quartile)” in the upper row, and “mean rank” in the lower row in  ^∗^3. Multiple comparisons (Bonferroni):  ^∗∗^ *p* < 0.01. Adjusted standard deviation: †, an absolute value of 1.96 or greater was considered significant. Physical activity was classified as low (< 10 MET‐hours/week), moderate (10.0–49.9 MET‐hours/week), and high (> 50 MET‐hours/week).

Abbreviations: Cl, cluster; n.s., not significant.

^∗^ *p* < 0.05;  ^∗∗^ *p* < 0.01;  ^∗∗∗^ *p* < 0.001.

For subjective well‐being, hobbies, physical pain, frailty, low physical activity, and number of physical activity items, additional analyses were performed. A significant difference was found in frailty (*p* = 0.005), and residual analysis indicated that the proportion of frailty was significantly lower in the “aerobic exercise group” and significantly higher in the “recreational gymnastics group.”

Next, a significant difference was observed in the amount of physical activity (*p* < 0.001). Residual analysis revealed that the “shopping group” and “recreational gymnastics group” had significantly higher percentages of low scores, whereas the “balance‐oriented group” had a significantly lower percentage of low scores. Furthermore, a significant difference was observed in the number of physical activity items (*p* < 0.001), and multiple comparisons showed that the “sports group” and “recreational gymnastics group” were significantly less active than the other groups. However, no significant differences were observed between clusters for subjective well‐being, enjoyment of activities, or physical pain (*p* > 0.05) (Table 4).

## 5. Discussion

In this study, we calculated the composition ratio of the physical activity categories using the OTBPAQ and examined their characteristics. In this section, we will discuss the distribution of physical activity of the participants, characteristics of OTBPAQ, and cluster classification, which was the main result of this study, as well as the limitations of this study.

### 5.1. Distribution of Physical Activity

The study participants were predominantly females, with no age difference between the sexes. The Ministry of Health, Labour and Welfare [16] recommended that the distribution of physical activity was 10 METs per hour/week. This is the standard value for physical activity for persons aged 65 years and over. This value was achieved by more than 80% of the older participants, suggesting that most of them had certain physical exercise habits.

Additionally, the OTBPAQ used in this study extracts physical activity items with an exercise load of ≥ 3 MET‐hours/week. Adding low‐intensity exercise items of < 3 MET‐hours/week is expected to increase physical activity. Therefore, the target population for this study consisted of active individuals.

In interpreting our findings, it is important to acknowledge that the assignment of MET values relied primarily on the Older Adult Compendium of Physical Activities [19]. This resource provides estimates that better reflect the lower resting metabolic rate in older adults than in young and middle‐aged adults, resulting in higher MET values in older adults than in younger adults. For activities not listed in the Older Adult Compendium, MET values were estimated by selecting the most similar activity through expert consensus among occupational therapists. While this approach ensured a consistent framework for estimating energy expenditure, it may introduce some degree of subjectivity. Nevertheless, the use of age‐appropriate MET values is particularly relevant. Older adults typically have a lower resting metabolic rate than younger adults. This means that the same activity may impose relatively greater physiological intensity in this population. Recognizing these differences is crucial for accurately interpreting physical activity patterns and designing interventions tailored to older adults’ functional capacities.

### 5.2. Characteristics of the OTBPAQ

Various physical activity questionnaires have been developed [20]. The most commonly used questionnaires in Japan and abroad are the International Physical Activity Questionnaire developed by Craig et al. [16, 21] and the Global Physical Activity Questionnaire developed by the World Health Organization [22]. Both questionnaires generally ask participants to reflect backward on their daily lives and indicate the time and frequency of physical activity by intensity or domain. The OTBPAQ used in this study was similar to these questionnaires in that it asked about the time and frequency, but differed significantly in presenting 16 physical activity items as prior information. Although this has the disadvantage of the answers being strongly influenced by the options provided, it assists the examinee’s recall. This can significantly reduce the ambiguity of memory regarding whether the listed physical activity was performed. Although the influence of prior information can be seen in the results of this study, it reduced the possibility that the test participants gave up recalling the information or underestimated the amount of physical activity without recalling it as an unconscious behavior. However, the validity of the OTBPAQ developed in this study requires an analysis of its criterion‐related validity using objective methods, such as accelerometers and the doubly labeled water.

### 5.3. Cluster Classification and Demographic Characteristics

In this study, the physical activity categories obtained from the OTBPAQ survey results were categorized based on occupational therapy physical activities. Five clusters were identified using cluster analysis based on the percentage of physical activity in each category.

The “sports group” consisted mainly of males (males: 31.0%) and included participants with relatively high physical activity levels (≥ 50 MET‐hours/week: 31.0%) and a greater number of physical activity items (range: 3–6). This may reflect gender‐related preferences and social factors that influence engagement in structured exercise or sports activities. In contrast, the “shopping group” had a higher proportion of females and frail individuals (females: 89.7%; frailty: 30.4%), showing a lower proportion of participants with high physical activity levels (≥ 50 MET‐hours/week: 11.2%) and fewer physical activity items (range: 2–4). Shopping represents an essential IADL activity, often retained even among older adults with limited functional capacity. Therefore, this group may represent individuals performing only the minimum level of necessary physical activity in daily life.

The “balance‐oriented” and “aerobic exercise” groups showed relatively high physical activity levels (≥ 50 MET‐hours/week: 30.0% and 29.3%, respectively) and a lower prevalence of frailty (frailty: 38.9% and 30.4%, respectively). The results of the aerobic exercise group suggest that engaging regularly in moderate‐intensity physical activity contributes to maintaining functional independence and overall health [6]. Meanwhile, the findings from the “balance‐oriented group” showed that participants engaged in a diverse physical activity items (range: 4–6), suggesting that performing various physical activities in a well‐balanced manner contributes to maintaining both physical and mental health [8, 9]. In contrast, the “recreational gymnastics group” primarily engaged in low‐intensity exercises (mean: 3.6 METs), which accounted for 87% of their total physical activity composition. This group performed fewer physical activity items (range: 1–3), had a higher prevalence of frailty (frailty: 73.3%), and included no participants with high physical activity levels (≥ 50 MET‐hours/week: 0%). This finding suggests potential issues related to insufficient exercise intensity, limited duration, and lack of activity diversity. For maintaining functional independence and overall health, it is important to monitor exercise intensity and duration, while encouraging engagement in a variety of physical activities in a balanced manner [8, 9]. These demographic differences imply that physical activity patterns in older adults are shaped not only by physical capability but also by social roles, gender norms, and opportunities for participation [11]. Tailoring interventions according to these demographic profiles could enhance the effectiveness of community‐based health promotion.

### 5.4. Necessity and Implications of the Composition Ratio of Physical Activity Categories in Relation to Total Daily Physical Activity

Increased physical activity is essential for extending healthy life expectancy and preventing physical inactivity among older adults [23]. However, the intensity and duration of physical activity of an individual are limited by various factors, including both individual and environmental factors. Therefore, it is necessary to consider the composition ratio of physical activity categories in relation to total daily physical activity [24]. To calculate the amount of physical activity required for each individual or to specify the types of physical activity, a tailor‐made approach that matches the individual’s life stage and lifestyle with respect to the composition ratio of physical activity types would be practical. For example, physical activity positively affects physical factors, such as muscle strength and joint flexibility; mental and psychological factors, such as dementia and depression; and social factors, such as living alone and economic deprivation [6]. Our results suggest that the composition ratio of the physical activity categories in the analyzed clusters influences various participant characteristics. Therefore, assessing the composition ratio of physical activity categories may help identify appropriate activity types for each individual when promoting initiatives to extend healthy life expectancy.

From a clinical perspective, this approach allows occupational therapists and healthcare professionals to design individualized intervention programs. By understanding which activity categories are dominant or lacking in each individual, clinicians can recommend adjustments that promote balanced physical engagement, enhance endurance, and prevent frailty. For example, those with limited diversity of activities—such as individuals in the “recreational gymnastics group”—could benefit from gradual increases in activity intensity or variety to improve overall functional capacity.

From a social and community perspective, evaluating the composition ratio of physical activity categories in relation to total daily physical activity can inform community‐based interventions and public health policies. Local authorities and welfare organizations can design exercise and participation programs that align with the actual lifestyle patterns of older adults in their area, such as integrating moderate‐intensity physical activities into daily routines or creating opportunities for social participation through group‐based exercises. These strategies may contribute not only to reducing frailty but also to enhancing social connectedness and quality of life among community‐dwelling older adults.

Therefore, in occupational therapy practice, the results of this study can serve as a useful indicator for assessing balance in physical activity composition and providing individualized feedback. This perspective emphasizes that maintaining a well‐balanced combination of physical, mental, and social activities is essential for sustaining health and independence in later life.

### 5.5. Limitations of This Study

This study was conducted among older adults attending a community center for older individuals. This group was active and interacted with others, and a large proportion of the participants were females. Therefore, the composition of physical activity categories may differ from those who do not attend senior citizen community centers. In addition, the accuracy of the OTBPAQ in recalling the amount of physical activity must be clarified. As with the existing backward‐looking self‐administered questionnaires, the amount of physical activity recalled on the OTBPAQ may vary depending on how each individual perceives and remembers physical activity. In the future, we would like to clarify the characteristics and accuracy of the OTBPAQ by comparing it with other self‐administered questionnaires and determine its relevance to “objective assessment” using physical activity monitors and pedometers. Furthermore, there is significant individual variation in the physical function (mobility, muscle strength, and endurance) of older adults. These differences influence the types and intensity of physical activities older adults can perform, potentially explaining some of the cluster patterns observed in this study. Examining the relationship between OTBPAQ and physical function is expected to improve classification accuracy and interpretation.

### 5.6. Conclusions

In this study, we calculated the composition ratios of physical activity categories in relation to total daily physical activity using the OTBPAQ and examined the characteristics of five clusters of physical activity categories. The participant characteristics of these clusters were observed to differ. Most participants engaged in lower intensity balance‐oriented physical activities (49.5%), and the fewest engaged in higher intensity recreational gymnastics physical activities (3.0%). The results of this study suggest that the “composition ratio of physical activity categories” facilitates a highly individualized approach to physical activity, which can help implement more effective measures that contribute to an increase in healthy life expectancy.

## Conflicts of Interest

The authors declare no conflicts of interest.

## Funding

No funding was received for this manuscript.

## Data Availability

The data that support the findings of this study are available from the corresponding author upon reasonable request.

## References

[1] Lee I. M. , Sesso H. D. , Oguma Y. , and Paffenbarger R. S.Jr., Relative Intensity of Physical Activity and Risk of Coronary Heart Disease, Circulation. (2003) 107, no. 8, 1110–1116, 10.1161/01.CIR.0000052626.63602.58, 2-s2.0-0037418222. 12615787

[2] Sesso H. D. , Paffenbarger R. S.Jr., and Lee I. M. , Physical Activity and Coronary Heart Disease in Men, Circulation. (2000) 102, no. 9, 975–980, 10.1161/01.CIR.102.9.975, 2-s2.0-0034730069. 10961960

[3] Carter N. D. , Kannus P. , and Khan K. M. , Exercise in the Prevention of Falls in Older People, Sports Medicine. (2001) 31, no. 6, 427–438, 10.2165/00007256-200131060-00003, 2-s2.0-0035000557. 11394562

[4] Brach J. S. , Simonsick E. M. , Kritchevsky S. , Yaffe K. , Newman A. B. , and for the Health, Aging and Body Composition Study Research Group , The Association Between Physical Function and Lifestyle Activity and Exercise in the Health, Aging and Body Composition Study, Journal of the American Geriatrics Society. (2004) 52, no. 4, 502–509, 10.1111/j.1532-5415.2004.52154.x, 2-s2.0-1842632474. 15066063

[5] Ramsbottom R. , Ambler A. , Potter J. , Jordan B. , Nevill A. , and Williams C. , The Effect of 6 Months Training on Leg Power, Balance, and Functional Mobility of Independently Living Adults Over 70 Years Old, Journal of Aging and Physical Activity. (2004) 12, no. 4, 497–510, 10.1123/japa.12.4.497, 2-s2.0-5444264024, 15851822. 15851822

[6] Zhao W. , Hu P. , Sun W. , Wu W. , Zhang J. , Deng H. , Huang J. , Ukawa S. , Lu J. , Tamakoshi A. , and Liu X. , Effect of Physical Activity on the Risk of Frailty: A Systematic Review and Meta-Analysis, PLoS One. (2022) 17, no. 12, e0278226, 10.1371/journal.pone.0278226, 36454790. 36454790 PMC9714708

[7] Ministry of Health, Labour and Welfare [online]. Physical Activity Standards for *Health Promotion*, 2013 [cited 2024 March 25] Available from: https://www.mhlw.go.jp/stf/houdou/2r9852000002xple.html.

[8] De Labra C. , Guimaraes-Pinheiro C. , Maseda A. , Lorenzo T. , and Millán-Calenti J. C. , Effects of Physical Exercise Interventions in Frail Older Adults: A Systematic Review of Randomized Controlled Trials, BMC Geriatrics. (2015) 15, no. 1, 10.1186/s12877-015-0155-4, 2-s2.0-84949477734, 26626157. PMC4667405 26626157

[9] Cadore E. L. , Rodríguez-Mañas L. , Sinclair A. , and Izquierdo M. , Effects of Different Exercise Interventions on Risk of Falls, Gait Ability, and Balance in Physically Frail Older Adults: A Systematic Review, Rejuvenation Research. (2013) 16, no. 2, 105–114, 10.1089/rej.2012.1397, 2-s2.0-84876581280, 23327448. 23327448 PMC3634155

[10] Kim S. , Park J. L. , Hwang H. S. , and Kim Y. P. , Correlation Between Frailty and Cognitive Function in Non-Demented Community Dwelling Older Koreans, Korean Journal of Family Medicine. (2014) 35, no. 6, 309–320, 10.4082/kjfm.2014.35.6.309, 2-s2.0-84912001639, 25426279. 25426279 PMC4242909

[11] Hoogendijk E. O. , Suanet B. , Dent E. , Deeg D. J. , and Aartsen M. J. , Adverse Effects of Frailty on Social Functioning in Older Adults: Results From the Longitudinal Aging Study Amsterdam, Maturitas. (2016) 83, 45–50, 10.1016/j.maturitas.2015.09.002, 2-s2.0-84952638166, 26428078. 26428078

[12] Statistics Bureau , Ministry of Internal Affairs and Communications [online]. Basic Survey of Social Life in 2021: Results on Lifestyle Behavior Available from: https://www.stat.go.jp/data/shakai/2021/pdf/gaiyoua.pdf.

[13] Ministry of Health, Labour and Welfare [online] , Utilization of Kihon Checklist, 2005 [cited 2024 March 25] Available from: https://www.mhlw.go.jp/topics/kaigo/kaigi/051219/dl/2.pdf.

[14] World Federation of Occupational Therapists [online] , About occupational therapy, 2012 [cited 2024 March 25] Available from: https://www.wfot.org/about-occupational-therapy.

[15] Boop C. , Cahill S. M. , Davis C. , Dorsey J. , Gibbs V. , Herr B. , Kearney K. , Metzger L. , Miller J. , Owens A. , and Rives K. , Occupational Therapy Practice Framework: Domain and Process-Fourth Edition, American Journal of Occupational Therapy. (2020) 74, no. Supplement_2, 1–85, 10.5014/ajot.2020.74S2001. 34780625

[16] Craig C. L. , Marshall A. L. , Sjöström M. , Bauman A. E. , Booth M. L. , Ainsworth B. E. , Pratt M. , Ekelund U. L. , Yngve A. , Sallis J. F. , and Oja P. , International Physical Activity Questionnaire: 12-Country Reliability and Validity, Medicine and Science in Sports and Exercise. (2003) 35, no. 8, 1381–1395, 10.1249/01.MSS.0000078924.61453.FB, 2-s2.0-0042855872, 12900694. 12900694

[17] Satake S. , Senda K. , Hong Y. J. , Miura H. , Endo H. , Sakurai T. , Kondo I. , and Toba K. , Validity of theKihon Checklist for Assessing Frailty Status, Geriatrics & Gerontology International. (2016) 16, no. 6, 709–715, 10.1111/ggi.12543, 2-s2.0-84971441400. 26171645

[18] Ogawa K. , Fujiwara Y. , Yoshida H. , Nishi M. , Fukaya T. , Kim M. , Amano H. , Lee S. , Watanabe N. , and Shinkai S. , The Validity of the “Kihon Check-List” as an Index of Frailty and Its Biomarkers and Inflammatory Markers in Elderly People, Nihon Ronen Igakkai zasshi. Japanese Journal of Geriatrics. (2011) 48, no. 5, 545–552, 10.3143/geriatrics.48.545, 2-s2.0-85009561274, 22323034. 22323034

[19] Willis E. A. , Herrmann S. D. , Hastert M. , Kracht C. L. , Barreira T. V. , Schuna J. M.Jr., Cai Z. , Quan M. , Conger S. A. , Brown W. J. , and Ainsworth B. E. , Older Adult Compendium of Physical Activities: Energy Costs of Human Activities in Adults Aged 60 and Older, Journal of Sport and Health Science. (2024) 13, no. 1, 13–17, 10.1016/j.jshs.2023.10.007, 38242593. 38242593 PMC10818108

[20] Nakata Y. , Sasai H. , Murakami H. , Kawakami R. , Tanaka S. , and Miyachi M. , Scoring Protocol for Calculation of Total Energy Expenditure by Physical Activity Questionnaires Used in Japanese Cohort Studies, Research in Exercise Epidemiology. (2017) 19, no. 2, 83–92.

[21] Murase N. , Katumura T. , Ueda T. , Inoue S. , and Shimomistu T. , International Standardization of the Physical Activity Energy; Reliability & Validity Evaluation of Japanese Version International Physical Activity Questionnaire (in Japanese), Index of Public Welfare. (2002) 49, 1–9.

[22] Bull F. C. , Maslin T. S. , and Armstrong T. , Global Physical Activity Questionnaire (GPAQ): Nine Country Reliability and Validity Study, Journal of Physical Activity & Health. (2009) 6, no. 6, 790–804, 10.1123/jpah.6.6.790, 2-s2.0-73349134603, 20101923. 20101923

[23] Inoue M. , Iso H. , Yamamoto S. , Kurahashi N. , Iwasaki M. , Sasazuki S. , Tsugane S. , and Japan Public Health Center-Based Prospective Study Group , Daily Total Physical Activity Level and Premature Death in Men and Women: Results From a Large-Scale Population-Based Cohort Study in Japan (JPHC Study), Annals of Epidemiology. (2008) 18, no. 7, 522–530, 10.1016/j.annepidem.2008.03.008, 2-s2.0-44949242353, 18504139. 18504139

[24] Westerterp K. R. , Assessment of Physical Activity: A Critical Appraisal, European Journal of Applied Physiology. (2009) 105, no. 6, 823–828, 10.1007/s00421-009-1000-2, 2-s2.0-63049091166. 19205725

